# NeuroSwipe: Crowdsourcing the Brain–Citizen Science in Neuroimaging Research

**DOI:** 10.1192/bjo.2025.10100

**Published:** 2025-06-20

**Authors:** Ana Mirza-Davies, Sonya Foley, Derek Jones, Judith Harrison

**Affiliations:** ^1^Imperial College CR&T Dementia Research Institute, London, United Kingdom; ^2^Cardiff University, Cardiff, United Kingdom; ^3^Newcastle University, Newcastle, United Kingdom

## Abstract

**Aims:** Large-scale neuroimaging projects rely on automated pipelines to reconstruct white matter tracts from diffusion MRI (dMRI) data. However, these reconstructions are not always accurate and often require labour-intensive manual review to identify artefacts. To address this challenge and extend the reach of science engagement beyond traditionally accessible groups, we developed NeuroSwipe: a web-based platform designed to involve the public in evaluating the quality of dMRI data. This initiative also aimed to enhance participants’ understanding of brain imaging techniques and the scientific process, fostering broader public involvement in research.

**Methods:** The initial concept was developed by a multidisciplinary team of computational neuroscientists, physicists, science engagement specialists, and clinical researchers. The NeuroSwipe prototype was created by students at the National Software Academy, Cardiff University, and co-designed with ten citizen scientists during a co-production workshop held at the Cardiff University Brain Research Imaging Centre (CUBRIC). The platform included a short interactive training module to guide participants. Users were tasked with approving or rejecting anonymised dMRI images based on their quality. To ensure diverse participation, we partnered with Diverse Cymru, a charity that facilitated engagement with BAME and traditionally harder-to-reach populations. User decisions were recorded and compared with expert classifications. A post-test questionnaire assessed usability, knowledge gains, and engagement.

**Results:** A total of 89 individuals, identified through community organisations, tested the NeuroSwipe platform over three months. Of these, 82 completed the training module before rating images. Classifications by citizen scientists showed high consistency with expert evaluations, with no significant differences observed. Post-test feedback indicated that 72% of participants found the platform ‘easy’ or ‘very easy’ to use, and 63% thought the training module provided ‘about the right amount of information’, although 9% felt it was insufficient. Importantly, 92% described the platform as ‘engaging’ or ‘informative’. Free-text comments revealed increased understanding of brain imaging techniques and a sense of contribution to scientific research. The project was later publicised by BBC News and Wales Online, further amplifying its reach.

**Conclusion:** This project highlights the potential of engaging citizen scientists in neuroimaging research through a web-based platform like NeuroSwipe. The findings demonstrate that citizen scientists can meaningfully contribute to assessing dMRI data quality while enhancing their understanding of brain imaging research. Future developments could include scaling the platform to incorporate other imaging modalities and integrating more advanced training modules to further expand public participation in neuroimaging research.

